# Increased bovine Tim-3 and its ligand expressions during bovine leukemia virus infection

**DOI:** 10.1186/1297-9716-43-45

**Published:** 2012-05-23

**Authors:** Tomohiro Okagawa, Satoru Konnai, Ryoyo Ikebuchi, Saori Suzuki, Tatsuya Shirai, Yuji Sunden, Misao Onuma, Shiro Murata, Kazuhiko Ohashi

**Affiliations:** 1 Department of Disease Control, Graduate School of Veterinary Medicine, Hokkaido University, Sapporo, 060-0818, Japan; 2 Department of Veterinary Clinical Sciences, Graduate School of Veterinary Medicine, Hokkaido University, Sapporo, 060-0818, Japan

## Abstract

The immunoinhibitory receptor T cell immunoglobulin domain and mucin domain-3 (Tim-3) and its ligand, galectin-9 (Gal-9), are involved in the immune evasion mechanisms for several pathogens causing chronic infections. However, there is no report concerning the role of Tim-3 in diseases of domestic animals. In this study, cDNA encoding for bovine Tim-3 and Gal-9 were cloned and sequenced, and their expression and role in immune reactivation were analyzed in bovine leukemia virus (BLV)-infected cattle. Predicted amino acid sequences of Tim-3 and Gal-9 shared high homologies with human and mouse homologues. Functional domains, including tyrosine kinase phosphorylation motif in the intracellular domain of Tim-3 were highly conserved among cattle and other species. Quantitative real-time PCR analysis showed that bovine Tim-3 mRNA is mainly expressed in T cells such as CD4^+^ and CD8^+^ cells, while Gal-9 mRNA is mainly expressed in monocyte and T cells. Tim-3 mRNA expression in CD4^+^ and CD8^+^ cells was upregulated during disease progression of BLV infection. Interestingly, expression levels for Tim-3 and Gal-9 correlated positively with viral load in infected cattle. Furthermore, Tim-3 expression level closely correlated with up-regulation of IL-10 in infected cattle. The expression of IFN-γ and IL-2 mRNA was upregulated when PBMC from BLV-infected cattle were cultured with Cos-7 cells expressing Tim-3 to inhibit the Tim-3/Gal-9 pathway. Moreover, combined blockade of the Tim-3/Gal-9 and PD-1/PD-L1 pathways significantly promoted IFN-γ mRNA expression compared with blockade of the PD-1/PD-L1 pathway alone. These results suggest that Tim-3 is involved in the suppression of T cell function during BLV infection.

## Introduction

In humans and mice, T cell immunoglobulin domain and mucin domain-3 (Tim-3), a member of the Tim family has been identified as a transmembrane protein, which contains an immunoglobulin and a mucin-like domain and is expressed on various cells such as CD4^+^Th1 cells, cytotoxic T-lymphocytes (CTL), monocytes and dendritic cells and natural killer cells, and characterized as a negative regulator for immune responses [1-3]. Tim-3 contains a predicted intracellular tyrosine-kinase phosphorylation motif, and acts as a functional receptor that transduces signals through the phosphorylated tyrosine residue [3,4]. Galectin-9 (Gal-9), a member of the Galectin family (also called S-type lectins) is secreted by several cells, and has been identified as the Tim-3 ligand [5]. Gal-9 consists of N- and C-terminal carbohydrate-binding domains, which are connected by a link peptide, and binds to Tim-3 via a carbohydrate chain [5-7]. Gal-9-induced intracellular calcium flux, aggregation, inhibition of cell proliferation and cytokine production as well as death of T cells are known to be Tim-3-dependent [5,8,9]. Tim-3 is expressed on activated T cells, including Th1 CD4^+^ and CD8^+^ cells resulting in selective loss of interferon (IFN)-γ producing cells and concomitant suppression of cell-mediated immunity. These observations suggest that the Tim-3/Gal-9 pathway may have evolved to ensure effective termination of effector Th1 cells [5].

Recently, several inhibitory receptor molecules including the Tim-3/Gal-9 pathway have been closely associated with immune exhaustion and disease progression in chronic infectious diseases and tumors [10-14]. Tim-3 is highly expressed on exhausted T-cells in lymphocytic choriomeningitis virus (LCMV) [15], Human Immunodeficiency Virus (HIV) [16], hepatitis C virus (HCV) infections [17,18] as well as in malignant melanoma [19]. Meanwhile, Gal-9 production from Kupffer cells is elevated during chronic HCV infection, and the elevated Gal-9 results in expansion of CD4^+^CD25^+^FoxP3^+^ regulatory T cells, contraction of CD4^+^ effector T cells, and apoptosis of HCV-specific CTL [20]. Thus, the Tim-3/Gal-9 pathway is one of the mechanisms in downregulating immune responses during chronic disease progression, and a potential target for novel immunotherapeutic intervention. However, there is no information available about the role of the Tim-3/Gal-9 pathway in bovine diseases.

Bovine leukemia virus (BLV), a retrovirus related to human T-cell leukemia virus type 1 (HTLV-1), causes enzootic bovine leucosis (EBL). Infection by BLV can remain clinically silent, with cattle in an aleukemic (AL) state, or it can emerge as a persistent lymphocytosis (PL), characterized by an increased number of B lymphocytes, and, more rarely, as B-cell lymphomas in various lymph nodes after a long latent period [21]. Recent work in our laboratory has revealed that inhibitory receptor molecules play a critical role in immune exhaustion and disease progression in BLV-infection [22-24], and blocking the inhibitory pathway in vitro increases cytokine responses and enhances function leading to a decrease in the viral load [23]. Therefore, such an evaluation of inhibitory receptor expression kinetics is essential to improve the design of an effective immunotherapy that can induce cell-mediated immune responses. In this study, to determine the possible role of bovine Tim-3 in chronic infectious diseases, we cloned both bovine Tim-3 and its ligand, Gal-9, and characterized their expression profiles using quantitative real-time PCR in cattle infected with BLV.

## Materials and methods

### Cloning of cDNA encoding for bovine Tim-3 and Gal-9

Bovine peripheral blood mononuclear cells (PBMC) were purified from heparinized venous blood of Holstein-Friesian breed maintained at the Graduate School of Veterinary Medicine, Hokkaido University, by density gradient centrifugation on Percoll solution (GE Healthcare UK Ltd., Amersham Place, Little Chalfont, Buckinghamshire, England). The PBMC were cultivated with 5 μg/mL Concanavalin A (Con A) (Sigma-Aldrich, St. Louis, MO, USA) for 12 h at 37°C in 5% CO_2_. Total RNA was isolated from cultivated PBMC using TRIzol reagent (Invitrogen, Carlsbad, CA, USA) according to the manufacturer’s instructions, and residual DNA was removed from the RNA samples by treatment with Deoxyribonuclease I (Invitrogen). cDNA was synthesized from 1 μg of total RNA with Moloney murine leukemia virus reverse transcriptase (Takara, Shiga, Japan) and the oligo-dT primer (0.5 mg/mL) according to the manufacturer’s instructions. To clone bovine Tim-3 and Gal-9 genes, specific primers were designed based on the sequences from Hereford breed cattle reported in the GenBank database (XM_001077105 and NM_001039177). Tim-3 cDNA was amplified by PCR using specific primers, 5′-AAA CGG CAC CTA AAC AGA GC-3′ and 5′-GAC AAC ACC AAG CCC CTA GA-3′. The cycling conditions consisted of initial denaturation at 94°C for 5 min, followed by 40 cycles of 94°C for 30 s, 55–47°C at the rate of 0.2°C/cycle for 1 min and 72°C for 1 min, and final extension at 72°C for 7 min. Gal-9 cDNA was amplified by PCR using specific primers, 5′-GGG AGA AGT GGC AGT GGC TAC AGA-3′ and 5′-ATC CAG ATA GCA GCA CAG GGC AG-3′. PCR condition was the same as above except annealing temperature (60–52°C). The PCR amplicons were purified by GENECLEAN III Kit (Q-Biogene, Carlsbad, CA, USA) and cloned into the pGEM-T Easy vector (Promega, Madison, WI, USA). The purified plasmid clones were sequenced using CEQ 2000 DNA Analysis System (Beckman Coulter, Fullerton, CA, USA). The established sequences were aligned, and an unrooted neighbor-joining tree was constructed using MEGA software program version 5.0 [25].

### Expression analysis of bovine Tim-3 and Gal-9 mRNA

CD4^+^, CD8^+^, CD5^+^, CD14^+^ and CD21^+^ cells were freshly isolated from bovine PBMC using BD IMagnet Cell Separation System (BD Biosciences, Franklin Lakes, NJ, USA) and the following antibodies: anti-bovine CD4 mouse monoclonal antibody (mAb) (CACT138T; VMRD, Pullman, WA, USA), anti-bovine CD8 mAb (IL-A51, a gift from the International Livestock Research Institute, Nairobi, Kenya), anti-bovine CD5 mAb (CACT105A, VMRD), anti-bovine CD14 mAb (CAM36A, VMRD) or anti-bovine CD21-like mAb (GB25A; VMRD), respectively. The purity of each cell population was confirmed with the EPICS XL flow cytometry system (Beckman Coulter) using EPICS EXPO32 ADC software (Beckman Coulter). Purified cells (> 90%) were used for expression analysis by quantitative real-time PCR. cDNA was synthesized from RNA samples as described above. Quantitative RT real-time PCR was performed with a LightCycler 480 system II (Roche Diagnostics, Mannheim, Germany) using SYBR premix DimerEraser (Takara) according to the manufacturer’s instructions. Primers were 5′-GGA TCC AAT TCC CAG GTC TAA-3′ and 5′-AGG GTC TTC AGT GTC CGT GT-3′ for Tim-3, 5′-ACA TCC GCG CAG CTC CCA AG-3′ and 5′-TTG ATT TGC GCC CCC TGG GC-3′ for Gal-9, and 5′-TCT TCC AGC CTT CCT TCC TG-3′ and 5′-ACC GTG TTG GCG TAG AGG TC-3′ for β-actin. The cycling conditions consisted of initial denaturation at 94°C for 30 s, followed by 45 cycles of 95°C for 5 s, 60°C for 30 s and 72°C for 30 s. A final melting curve analysis was performed from 65°C to 95°C at a rate of 0.1°C/s (continuous acquisition), with a final cooling to 40°C over 10 s. To estimate mRNA expression, calibration curves were made from the measured fluorescence of dilution series of the control plasmid to create the same amplification curves. Each sample was tested in duplicate, and the results of Tim-3 and Gal-9 mRNA expression are presented as a ratio obtained by dividing the concentrations of Tim-3 and Gal-9 mRNA by that of β-actin mRNA.

### BLV-infected cattle

To evaluate bovine Tim-3 and Gal-9 mRNA expression in infectious diseases, expression analyses were conducted with BLV-infected cattle diagnosed at the Veterinary Teaching Hospital, Graduate School of Veterinary Medicine, Hokkaido University, between 2007 and 2011. Genomic DNA was extracted from whole blood using the Wizard^TM^ Genomic DNA Purification kit (Promega). BLV infection was detected by nested-PCR, and the provirus load confirmed by real-time PCR as described previously [23]. Five cattle with lymphoma were diagnosed clinically and confirmed as EBL through microscopy and histological examinations. In other BLV-positive cattle, the numbers of leukocytes were counted using a Celltac α MEK-6450 automatic hematology analyzer (NIHON KOHDEN, Tokyo, Japan), and classified as AL (*n* = 14) or PL ( *n* = 10) as described previously [23]. Ten BLV-uninfected cattle were used as controls. To examine the degree of immunosuppression in BLV-infected cattle, IFN-γ and interleukin (IL)-10 mRNA expression levels in PBMC were quantified as described previously [26]. β-actin mRNA expression was also used as an internal control to normalize the target genes.

### Generation of bovine Tim-3 expressing cells and competitive assay

To generate Tim-3 expression vector, bovine Tim-3 cDNA was amplified as above except that primers with restriction enzyme recognition sites, Tim-3-FLAG-F (*EcoR*V): 5′-CGG CGA TAT CGC ATA TGT GTC TCA GGT G-3′, and Tim-3-FLAG-R ( *Not*I): 5′-ATT TGC GGC CGC TTA GGA TTG ATG CCC ACT-3′, were used instead. The amplified PCR product was digested with *EcoR*V(Takara) and *Not*I (TOYOBO, Osaka, Japan), and purified using Fast Gene Gel/PCR Exraction Kit (Nihon genetics, Tokyo, Japan). The purified product was ligated into the expression vector pCXN2.1-FLAG vector [27], kindly donated by Dr Takehiko Yokomizao, Graduate School of Medicine, Kyushu University, Fukuoka, Japan, and transformed into a competent DH5α *Escherichia coli* strain. The recombinant plasmid bearing Tim-3 in frame with a FLAG epitope tag was purified by QIAGEN Plasmid Mega kit, according to the manufacturer’s protocol (QIAGEN, Hilden, Germany), and introduced into Cos-7 cells (African Green Monkey SV40-transformed kidney fibroblast cell line) via transient transfection with Lipofectamine^TM^2000 (Invitrogen). To confirm Tim-3 expression on Cos-7 cells, western blot analysis and flow cytometry were performed using anti-FLAG M2 mouse monoclonal antibody (Sigma) and anti-DYKDDDDK mouse monoclonal antibody (TransGenic, Hyogo, Japan), respectively [28,29].

To investigate the influence of Tim-3 on cytokine production, PBMC (8 × 10^6^ cells) derived from BLV-infected cattle (*n* = 3) and Tim-3 expressing Cos-7 (1 × 10^6^ cells) were co-cultured in RPMI 1640 medium (Sigma-Aldrich) containing 10% FCS (Valley Biomedical, Winchester, VA, USA) and 1% L-glutamine (Invirtogen) at 37°C with 5% CO_2_. After 12 h, the PBMC were harvested and used to quantify cytokine expression. Furthermore, to confirm the synergistic effect of dual inhibitory molecule blockage, in the same plate, Con A (5 μg/mL), anti-human programmed death-ligand 1 (PD-L1) antibody (10 μg/mL, Santa Cruz Biotechnology, Santa Cruz, CA, USA) or IgG isotype control rabbit antibody (Beckman Coulter) were added to the co-cultivation set-ups at the beginning of culture and PBMC harvested after 12 h later as described above. To quantify cytokine mRNA expression, real-time quantitative RT-PCR for IL-2 and IFN-γ quantification were conducted as described previously [26].

### Statistics

Two-tailed unpaired Student’s *t*-test and two-tailed unpaired Welch’s  *t*-test were used for comparison tests and Spearman rank test was used for correlation analyses. *P* values of *p* < 0.05 were considered significant.

### Nucleotide sequence accession numbers

The sequences of the bovine Tim-3 and Gal-9 genes have been submitted to the GenBank database under accession number AB689695 (Tim-3) and AB689696 (Gal-9).

## Results

### Sequence analysis of bovine Tim-3 and its ligand

**Figure 1:**
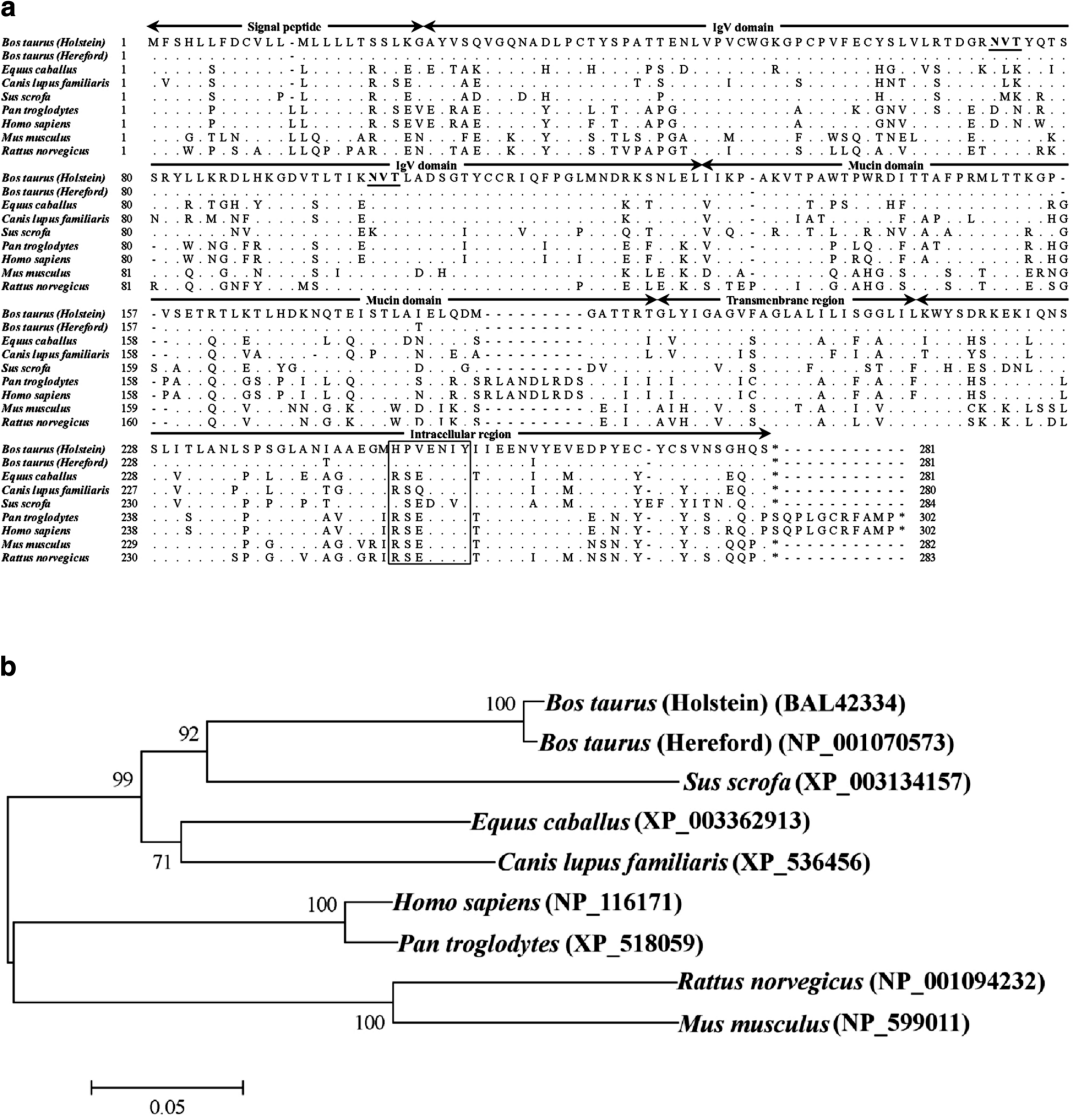
**Bovine Tim-3.(A)** Alignment of deduced amino acid sequences of Tim-3. Dots indicate identity to the sequence from Holstein breeds and dashes indicate gaps in alignment. Two potential N-glycosylation sites are shown in bold and underlined. The potential tyrosine kinase phosphorylation motif is indicated in the box. The accession numbers of analyzed sequences corresponded to those in Table 1. **(B)** A phylogenetic tree constructed based on the amino acid sequences of Tim-3. The tree was built with the neighbor-joining method using the MEGA 5.0 software. Numbers indicate bootstrap percentage (1000 replicates). The scale indicates the divergence time.

**Figure 2:**
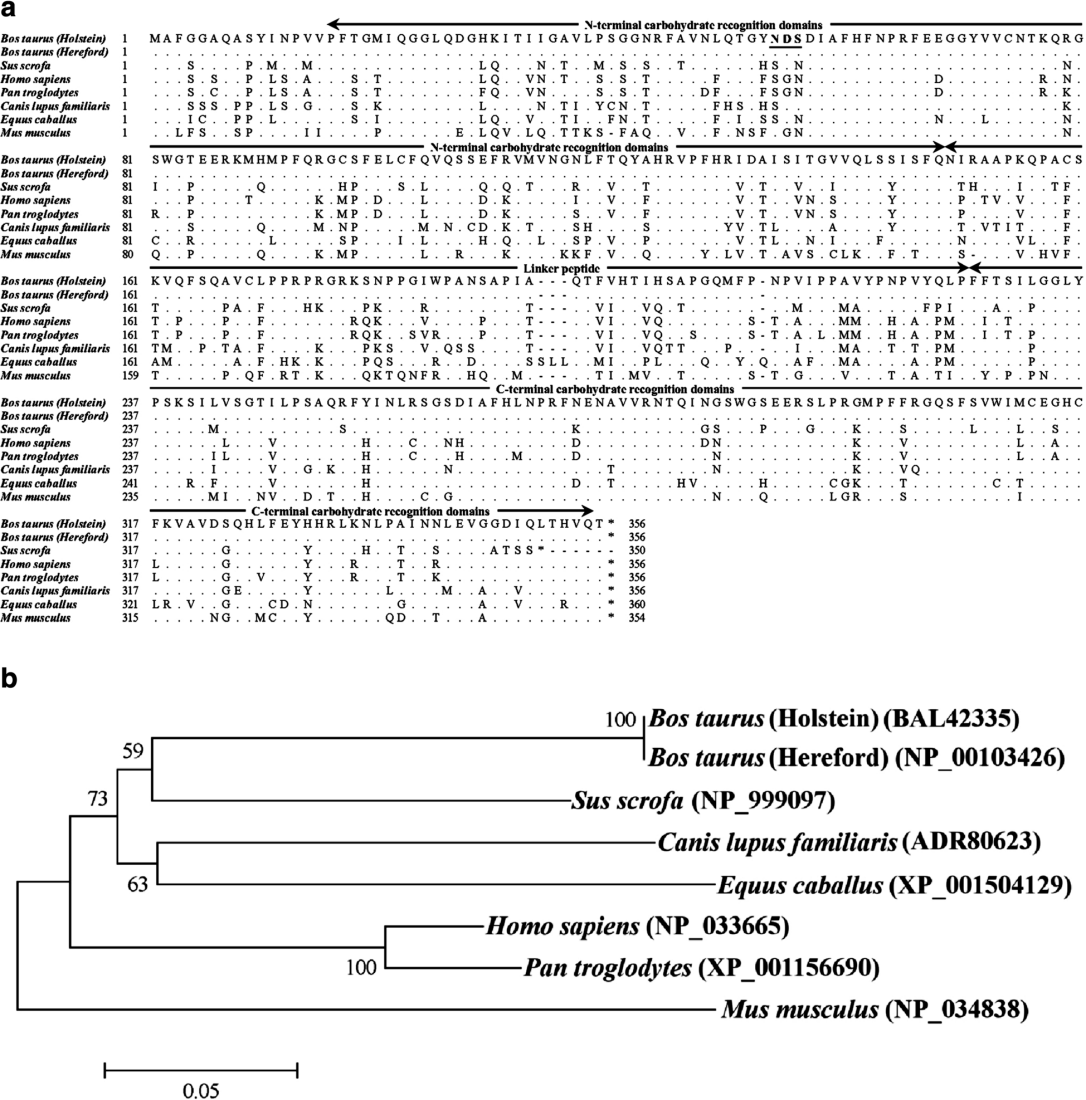
**Bovine Gal-9.** Alignment of deduced amino acid sequences of Gal-9 **(A)** and phylogenetic tree **(B)** were indicated as in Figure 1. The accession numbers of the analyzed genes corresponded to those in Table 2.

The putative amino acid sequence of bovine Tim-3 obtained from the Holstein breed is shown in Figure 1a. The bovine Tim-3 consists of a putative signal peptide of 23 amino acids (aa), the transmembrane domain of 23 aa and intracellular domain of 67 aa. Its extracellular domain, consisting of 168 aa, contains an immunoglobulin domain, the mucin domain and two potential N-linked glycosylation sites. The potential tyrosine kinase phosphorylation motif is conserved in all mammalian Tim-3 [30]. Phylogenetic analysis revealed that mammalian Tim-3 was divided into two groups, group 1 (Perissodactyla, Artiodactyla and Carnivora) and group 2 (Primate and Rodentia), with bovine Tim-3 clustering in the artiodactyl species group (Figure 1b). Comparative analysis of the Tim-3 sequences of several species showed that the bovine Tim-3 had amino acid identities of 75.8%, 74.7%, 73.3%, 69.3%, 69.0%, 62.8% and 59.9% with dog, horse, pig, human, chimpanzee, mouse and rat, respectively (Table 1). Sequence for Gal-9 obtained from the Holstein breed had an ORF encoding 356 amino acids (Figure 2a). Bovine Gal-9 consists of an N-terminal carbohydrate-recognition domain of 131 aa, a linker peptide of 80 aa and a C-terminal carbohydrate-recognition domain of 128 aa. The deduced Gal-9 amino acid sequence was further compared with those from the dog, horse, pig, human, chimpanzee and mouse (Table 2). The bovine Gal-9 had amino acid identities of 76.4%, 73.8%, 72.6%, 72.0%, 71.8% and 65.7% to the pig, human, chimpanzee, dog, horse and mouse, respectively. These results were further corroborated by phylogenetic analysis (Figure 2b), with the bovine Gal-9 falling into the larger clade of the Artiodactyla order.

**Table 1:** Similarity analysis of nucleic and amino acid sequences of Tim-3 among several animal species

	***Bos taurus*(Holstein)**	***Bos taurus*(Hereford)**	***Canis lupus familiaris***	***Equus caballus***	***Sus scrofa***	***Homo sapiens***	***Pan troglodytes***	***Mus musculus***	***Rattus norvegicus***
	**(AB689695)**	**(NM_001077105)**	**(XM_536456)**	**(NM_001077105)**	**(XM_003134109)**	**(NM_032782)**	**(XM_518059)**	**(NM_134250)**	**(NM_001100762)**
***Bos taurus*(Holstein)**	-	99.0	84.1	83.0	83.2	80.5	80.4	73.2	72.5
**(BAL42334)**									
***Bos taurus*(Hereford)**	98.9	-	84.7	83.4	83.8	80.6	80.8	73.6	73.1
**(NP_001070573)**									
***Canis lupus***	75.8	76.5	-	86.6	81.1	81.8	81.7	73.0	73.6
***familiaris***									
**(XP_536456)**									
***Equus caballus***	74.7	75.1	80.1	-	82.3	83.8	83.5	73.6	75.4
**(XP_003362913)**									
***Sus scrofa***	73.3	73.6	71.5	70.8	-	77.4	77.8	69.9	70.7
**(XP_003134157)**									
***Homo sapiens***	69.3	69.0	71.1	73.3	65.0	-	98.3	75.0	76.6
**(NP_116171)**									
***Pan troglodytes***	69.0	68.6	71.1	72.9	65.0	96.8	-	75.0	76.3
**(XP_518059)**									
***Mus musculus***	62.8	62.5	62.1	60.3	54.9	65.0	65.3	-	87.6
**(XP_518059)**									
***Rattus norvegicus***	59.9	60.3	63.2	61.4	56.7	65.7	66.1	81.2	-
**(NP_001094232)**									

Upper region shows percentages of homologies in nucleic acid level, and lower region shows those of deduced amino acid level. GenBank accession numbers are in parentheses after species names.

**Table 2:** Similarity analysis of nucleic and amino acid sequences of Gal-9 among several animal species

	***Bos taurus*(Holstein)**	***Bos taurus*(Hereford)**	***Sus scrofa***	***Homo sapiens***	***Pan troglodytes***	***Canis lupus familiaris***	***Equus caballus***	***Mus musculus***
	**(AB689696)**	**(NM_001039177)**	**(NM_213932)**	**(NM_009587)**	**(XM_001156690)**	**(HQ637390)**	**(XM_001504079)**	**(NM_010708)**
***Bos taurus*(Holstein)**	-	99.9	85.1	82.4	82.0	81.2	80.7	73.8
**(BAL42335)**								
***Bos taurus*(Hereford)**	100.0	-	85.0	82.3	81.9	81.1	80.7	73.7
**(NP_00103426)**								
***Sus scrofa***	76.4	76.4	-	82.8	82.2	82.2	81.1	74.8
**(NP_999097)**								
***Homo sapiens***	73.8	73.8	77.2	-	95.9	82.3	81.4	77.8
**(NP_033665)**								
***Pan troglodytes***	72.6	72.6	75.8	93.9	-	81.9	81.2	77.9
**(XP_001156690)**								
***Canis lupus familiaris***	72.0	72.0	74.6	74.9	73.2	-	80.8	73.7
**(ADR80623)**								
***Equus caballus***	71.8	71.8	71.8	71.8	71.5	72.6	-	74.8
**(XP_001504129)**								
***Mus musculus***	65.7	65.7	67.4	69.5	69.2	64.8	64.3	-
**(NP_034838)**								

Upper region shows percentages of homologies in nucleic acid level, and lower region shows those of deduced amino acid level. GenBank accession numbers are in parentheses after species names.

### Expression analysis of Tim-3 and its ligand during BLV disease progression

**Figure 3:**
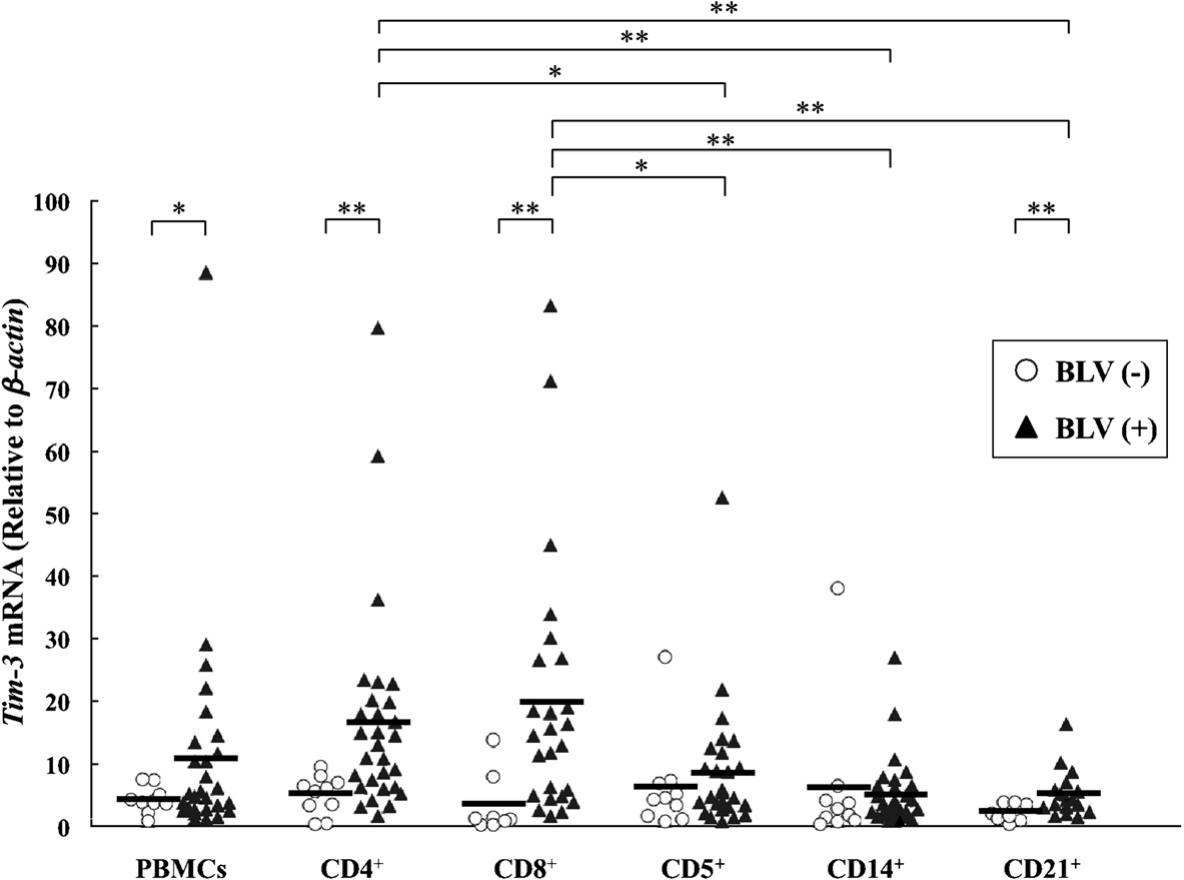
**Quantification of Tim-3 mRNA expression in PBMC and subpopulations of CD4**^**+**^**, CD8**^**+**^**, CD5**^**+**^**, CD14**^**+**^**and CD21**^**+**^**cells derived from BLV-uninfected and BLV-infected cattle using real-rime PCR analysis.** The concentration of Tim-3 mRNA was divided by that of β-actin mRNA. Each line indicates the mean percentages in each group. Asterisks donate significant differences between the types of the cells (* *p* < 0.05 and ** *p* < 0.01).

**Figure 4:**
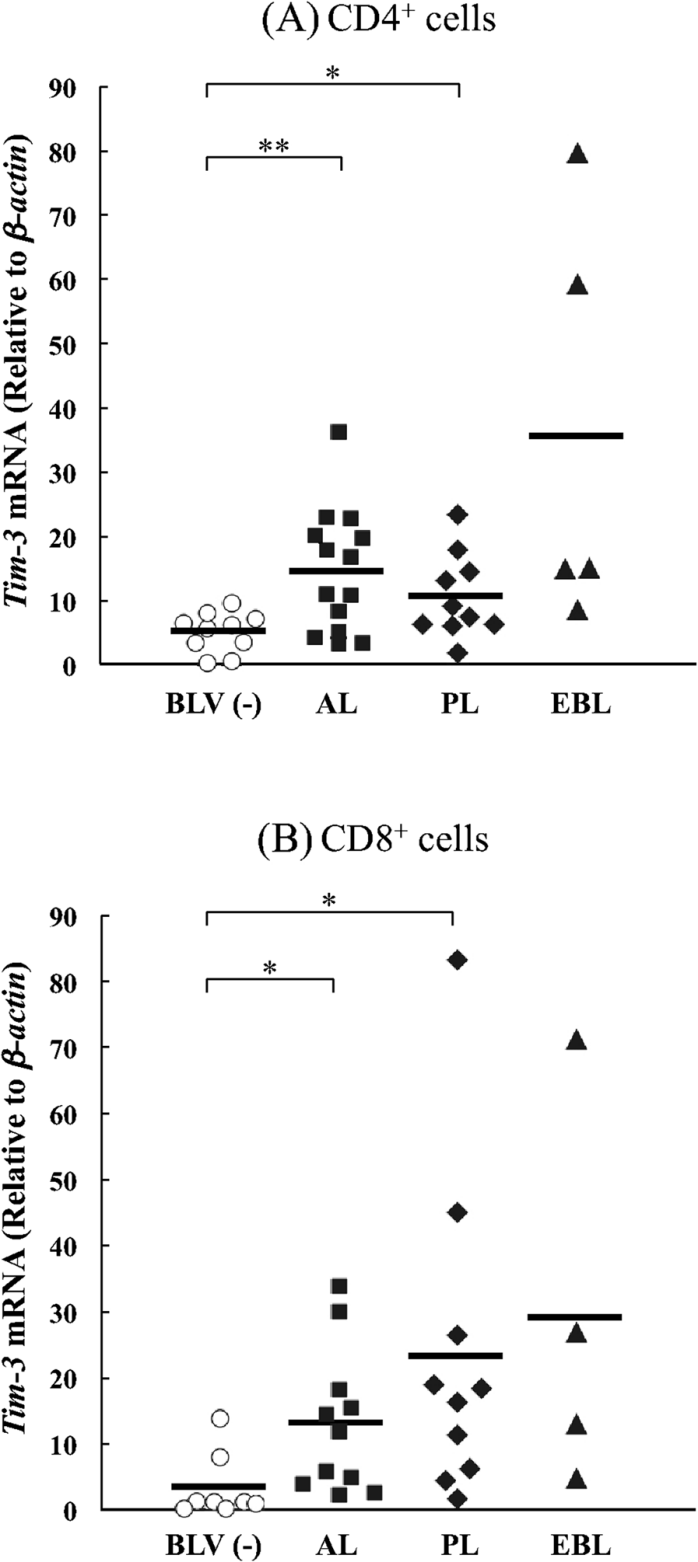
**Quantitative analysis of bovine Tim-3 expression in BLV-infected cattle at different disease stages.** CD4^+^**(A)** and CD8^+^**(B)** cells from BLV-negative and BLV-infected cattle with AL, PL and lymphoma (EBL) were analyzed. Individual dots indicate Tim-3 expression level in the cells from BLV-uninfected (empty circle), AL (full square), PL (full quarry) and lymphoma (full triangle). Each line indicates the mean percentages in each group. Differences between groups were considered statistically significant at probability values of *p* < 0.05 (* *p* < 0.05; ** *p* < 0.01).

**Figure 5:**
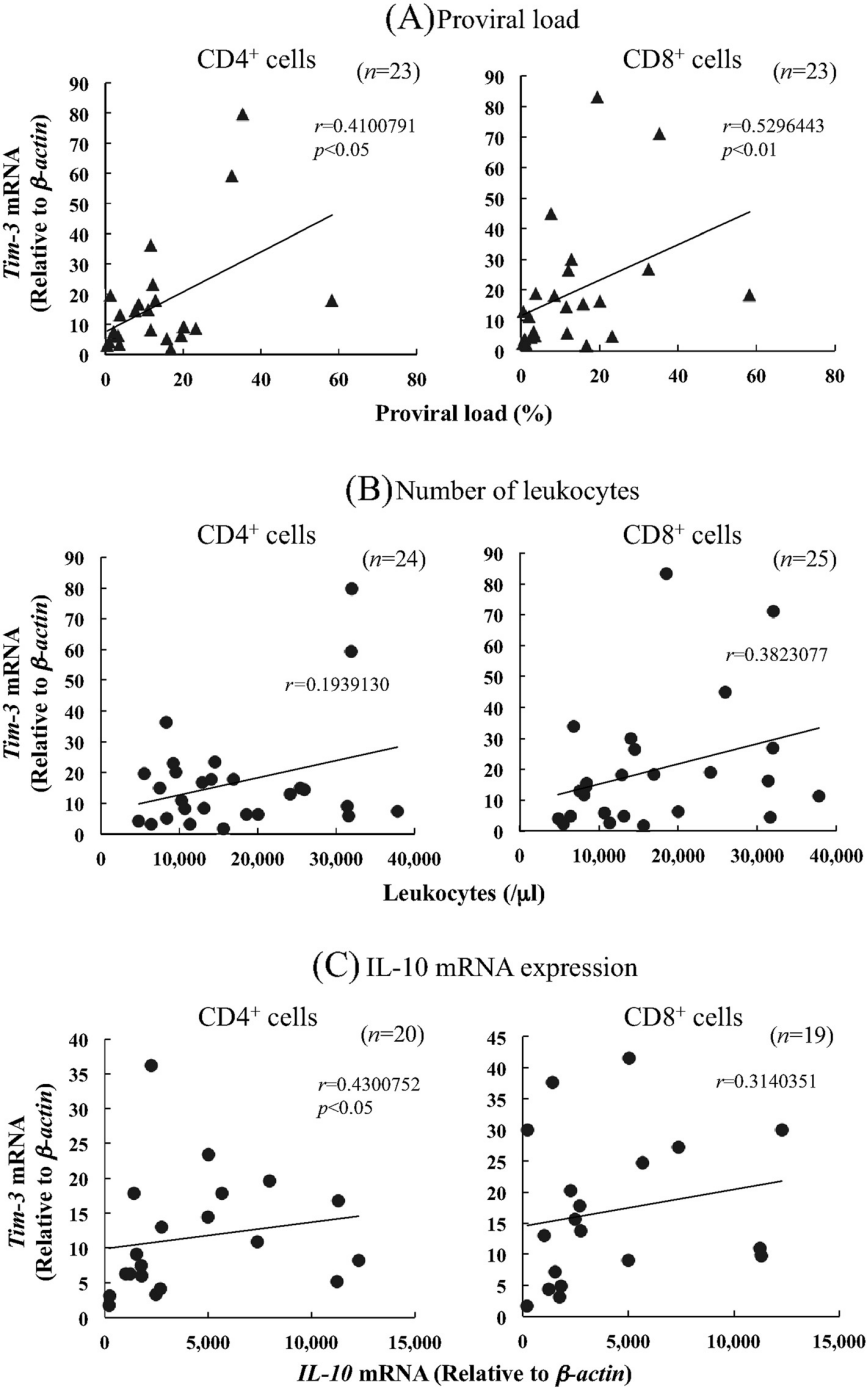
**Correlation between indicators of BLV disease progression and Tim-3 mRNA expression in BLV-infected cattle.(A)** Positive correlations were observed between proviral load ( *n* = 23) and Tim-3 expression in CD4^+^ cells and CD8+ cells. **(B)** Correlation between the leukocyte number and Tim-3 expression in BLV-infected cattle. **(C)** Correlation between IL-10 expression and Tim-3 expression in CD4^+^ cells was also positive. Proviral load and IL-10 mRNA expression were determined using quantitative real-time PCR analysis. Correlation statistics were analyzed by the Spearman rank test.

According to several reports based on a mouse model, Tim-3 is mainly expressed in dendritic cells (DC) and monocytes in healthy animals [1,31]. However, Tim-3 is upregulated in CD4^+^ and CD8^+^ T cells in LCMV and HIV chronic infections, and acts as a negative regulator of T cell functions [15,16]. To investigate the expression levels of bovine Tim-3 in bovine chronic infections, we first evaluated Tim-3 mRNA expression in several cell types among PBMC isolated from BLV-infected cattle using quantitative real-time PCR analysis (Figure 3). There were no significant differences in Tim-3 expression levels among the cell types from BLV-free cattle. Conversely, Tim-3 expression was significantly upregulated in PBMC from BLV-infected cattle compared to the uninfected cattle (*p* < 0.05). More specifically, Tim-3 expression levels in CD4^+^ and CD8^+^ T cells from infected cattle were significantly higher than in the control group (*p* < 0.01). In BLV-infected cattle, Tim-3 mRNA was mainly expressed in CD4^+^ and CD8^+^ T cells (Figure 3), with the expression levels in CD4^+^ T cells being significantly higher in aleukemic (AL) (*p* < 0.01) and persistent lymphocytosis (PL) ( *p* < 0.05) cattle compared to the uninfected animals (Figure 4a). Likewise, significant upregulation of Tim-3 mRNA was observed in CD8^+^ T cells from AL (*p* < 0.05) and PL ( *p* < 0.05) cattle (Figure 4b). Tim-3 expression levels correlated positively with viral load in CD4^+^ (*p* < 0.05) and CD8^+^ (*p* < 0.01) T cells in the infected cattle (Figure 5a). Furthermore, increased Tim-3 expression levels were associated with the number of leukocytes although the difference was statistically insignificant (Figure 5b). Tim-3 expressions in CD4^+^ and CD8^+^ T cells have a tendency to upregulate IL-10 mRNA expression (Figure 5c).

**Figure 6:**
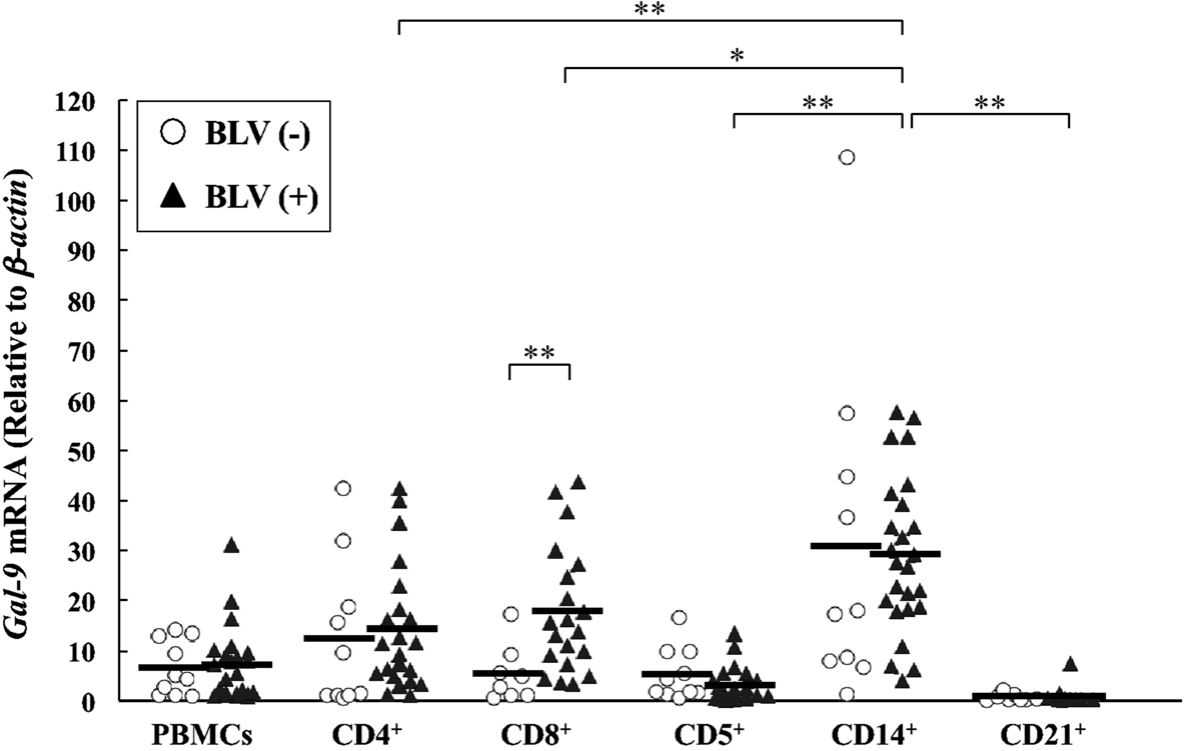
**Analysis of bovine Gal-9 mRNA expression in cells derived from BLV-uninfected and BLV-infected cattle.** The expression analysis was determined as in Figure 3. Each line indicates the mean percentages in each group. Asterisks donate significant differences between the types of the cells (*p < 0.05 and ** *p* < 0.01).

**Figure 7:**
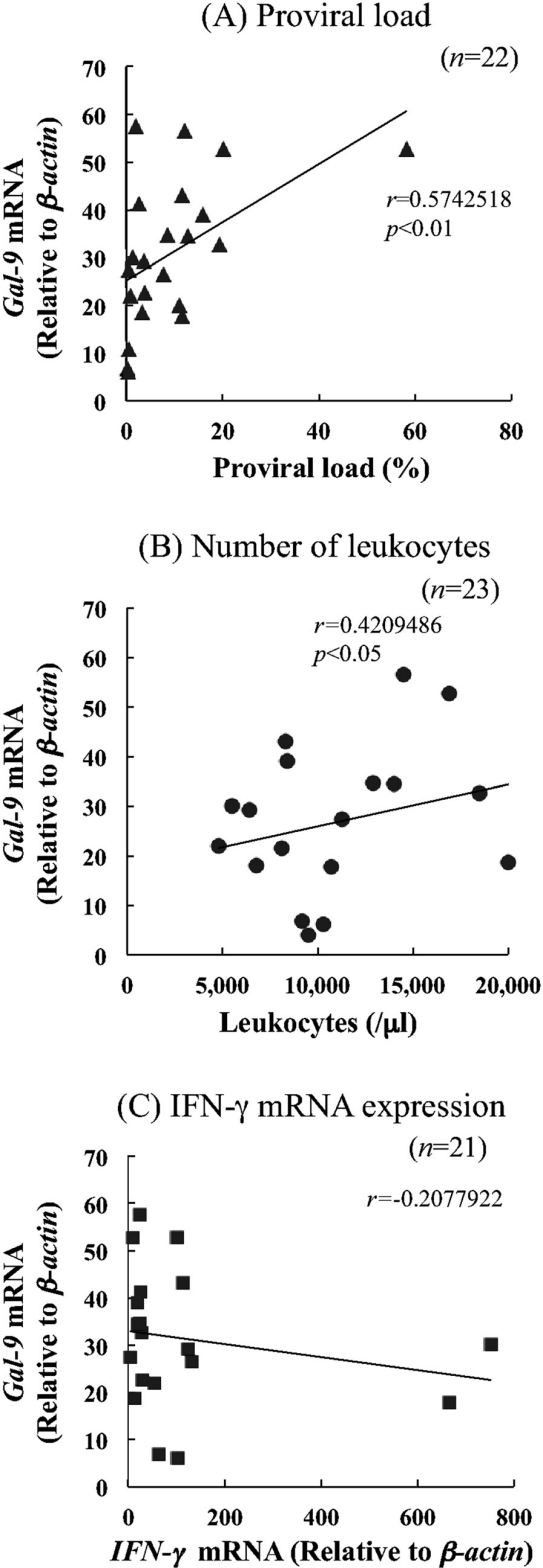
**Correlation between indicators of BLV disease progression and Gal-9 mRNA expression in CD14**^**+**^**cells from BLV-infected cattle.** Positive correlations were observed between proviral load **(A)**, number of leukocytes **(B)** and Gal-9 expression in CD14^+^ cells from the infected cattle. **(C)** Correlation between the IFN-γ expression and Gal-9 expression in BLV-infected cattle. Correlation statistics were analyzed by Spearman rank test.

Consequently, expression of Gal-9, the ligand for Tim-3, was also evaluated in BLV-infected cattle. Gal-9 mRNA was mainly expressed in CD14^+^ cells whereas its expression level in CD8^+^ T cells was significantly higher in BLV-infected cattle than uninfected ones (Figure 6). Significant positive correlations were noted between Gal-9 expression in CD14^+^ cells and proviral load (*p* < 0.01) (Figure 7a), and with the numbers of leukocytes (*p* < 0.05) (Figure 7b), suggesting that Gal-9 expression increased with BLV-disease progression. Gal-9 expression in CD14^*+*^ cells has a tendency to down-regulate IFN-γ mRNA expression, although there were no significant differences (Figure 7c).

### Effect of the Tim-3 inhibition on cytokine expression

**Figure 8:**
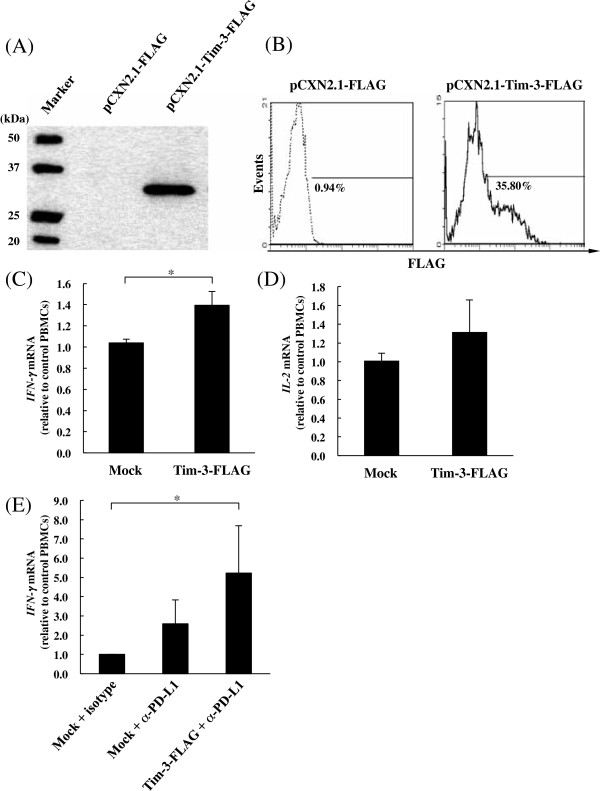
**Inhibition of the Tim-3/Gal-9 pathway increases cytokine expression in PBMC from BLV-infected cattle.** (A and B) The expression of bovine Tim-3 on Cos7 cells. The detections were performed by **(A)** Western blotting analysis and **(B)** Flowcytometric analysis with anti-FLAG antibody as described elsewhere. Empty plasmid-transfected cells were used as mock for each assay. (C, D and E) Real-time PCR quantification of mRNA expression levels for cytokines in the treated-PBMC from BLV-infected cattle ( *n* = 3). Up-regulations of IL-2 **(C)** and IFN-γ **(D)** expressions in PBMC by the inhibition of the Tim-3/Gal-9 pathway using Tim-3 expressing cells (Tim-3-FLAG). Up-regulations of IFN-γ **(E)** expressions by blockade of the PD-1/PD-L1 pathway using anti-PD-L1 antibody (α-PD-L1) and enhancement of the expression by combination PD-L1 blockade with Tim-3 expressing cells. Each cytokine mRNA level was normalized by the bovine β-actin mRNA as above, and the relative index was determined in comparison to the cytokine mRNA level in the PBMC without Tim-3 expressing cells and antibodies. The results are means of three independent experiments using cells from three individual cattle. Asterisks donate significant differences between the types of the cells (* *p* < 0.05).

To confirm recombinant expression of bovine Tim-3, a western blot assay and flow cytometric analysis were used to determine the expression of Tim-3 on Cos-7 cells. As shown in Figure 8a, bovine Tim-3 is detected as an approximately 29.9 kDa of a single species by Western blot analysis. Moreover, flow cytometry analysis confirmed that bovine Tim-3 was expressed on the surface of plasmid-transfected Cos-7 cells, with the proportion of Tim-3^+^ cells being 35.8% (Figure 8b).

To investigate the cause underlying the effects of Tim-3 on cytokine production, we examined the expression of anti-viral cytokine (IL-2 and IFN-γ) mRNA in cultured PBMC derived from BLV-infected cattle, in the presence of bovine Tim-3 expressing cells for trapping Tim-3 ligand, although a limited number of cattle were tested. As shown in Figure 8c, whereas the addition of Tim-3 expressing cells at the beginning of the 12-hr cultivation period clearly augmented IFN-γ level in bovine PBMC, the addition of mock plasmid-transfected cells had no effect on IFN-γ expression (*p* < 0.05). IL-2 mRNA was also increased by co-cultivation with the Tim-3 expressing cells, although this elevation was not statistically significant (Figure 8d). Recently, we demonstrated the enhancement of anti-viral immune responses in vitro via programmed death-1 (PD-1)/PD-L1 blockade in BLV infection [23]. Furthermore, to determine if the enhanced response can be up-regulated by Gal-9 trapping, PBMC from BLV-infected cattle were cultivated in the presence of anti-PD-L1 or isotype control antibody and Tim-3 expressing cells. As shown in Figure 8e, the addition of the Tim-3 expressing cells at the beginning of the 12-hr cultivation clearly enhanced IFN-γ expression in the PBMC.

## Discussion

Recently, the cases of BLV-induced EBL and infected cattle have been increasing in Japan, a worrisome trend given that there is no effective treatment and vaccination. The latter could be attributed to the lack of sufficient understanding of immunological mechanisms leading to immune evasion. During BLV-infection especially at the PL and lymphoma stages, T-cell dysfunction characterized by impaired cell proliferation and downregulation of Th1 cytokines accelerates disease progression through mechanisms yet to be elucidated [26,31-34]. In the present study, the genes for bovine Tim-3 and its ligand Gal-9, were cloned, sequenced, and subsequently, compared with sequences from various mammalian species. We also demonstrated the correlation between BLV infection and Tim-3 or Gal-9 expressions. Finally, a competitive assay using Tim-3 expressing cells for trapping Gal-9 was conducted to confirm that inhibition of Tim-3/Gal-9 pathway could lead to up-regulation of cytokine production in exhausted PBMC isolated from BLV-infected cattle. A deeper understanding of the Tim-3/Gal-9 pathway in domestic animals will facilitate the elucidation of events leading to immune dysfunction during the progression of incurable diseases including BLV-infection.

In our studies, bovine Tim-3 expression is widely observed in CD4^+^, CD5^+^, CD8^+^, CD14^+^ and CD21^+^ cells, consistent with similar findings in humans as well as mice [1,16]. Interestingly, Tim-3 expression in PBMC was found to be upregulated in BLV-infected cattle. In particular, the expression levels of Tim-3 in CD4^+^ and CD8^+^ T cells were significantly higher than those of other cell fractions, consistent with previous reports showing the upregulation of Tim-3 expression in chronic human diseases [16-19]. Upregulation of Tim-3 expression on anti-viral specific CD4^+^ and CD8^+^ T cells contributes to immune exhaustion, which results in increased viral load and disease progression in chronic infection with LCMV [15] and HCV [17,18]. Correspondingly, bovine Tim-3 mRNA expression in CD4^+^ and CD8^+^ cells was clearly upregulated with disease progression of BLV infection. Interestingly, Tim-3 expression levels correlated positively with viral load in the infected cattle. In addition, Tim-3 expression levels closely correlated with upregulation of IL-10, a negative regulatory cytokine, as observed in a previous study [33]. On the contrary, bovine Gal-9 is expressed in CD4^+^ cells, CD8^+^ cells and CD14^+^ cells as is in humans and mice [35,36]. The expression of Gal-9 in CD8^+^ T cells was significantly higher in BLV-infected cattle than uninfected ones. Interestingly, Gal-9 expression levels in CD14^+^ cells correlated positively with two markers of disease progression: leukocyte number and viral load. Gal-9 acts as a ligand of Tim-3, and induces cell death in Th1 cells but not in Th2 cells and cell death of Th1 cells via Tim-3 [5]. In addition, a previous study showed that in vivo administration of Gal-9 in immunized mice specifically reduced the numbers of IFN-producing Th1 cells [5]. Furthermore, functional studies suggest that Gal-9 induces expansion of CD4^+^CD25^+^FoxP3^+^ regulatory T cells and apoptosis of antigen specific CTL [20,37]. In the present study, Gal-9 expression correlated with downregulation of IFN-γ in the infected cattle, although this effect was statistically insignificant. These findings raise the possibility that the bovine Tim-3/Gal-9 pathway is also associated with T cell exhaustion in BLV-infection. However, Tim-3 expression levels did not correlate with downregulation of IFN-γ in contrast with our previous data [23], suggesting up-regulation of PD-L1 in BLV infected cattle (data not shown). In addition, the mechanism behind upregulation of Tim-3 and Gal-9 is still unclear. Further studies are necessary to determine the correlation between Tim-3 expression and T cell dysfunction in infected cattle.

Although the role of Tim-3/Gal-9 pathway in the immunosuppression of the BLV-infection is still speculative, the over-expression of Tim-3 and Gal-9 is apparently involved in T-cell dysfunctions during chronic disease progression in human and animal models. Recently, the blocking of Tim-3/Gal-9 pathway with an antibody specific to Tim-3 was shown to restore T cell function during HCV infection in vitro [18]. Moreover, in the cases of LCMV, HIV infection and cancer, Tim-3 and PD-1 are co-expressed by exhausted CD8^+^ T-cells, and dual blockade of Tim-3 and PD-L1 enhances T-cell responses as well as controlling virus and tumor [15,16,19,38]. These observations collectively indicate that Tim-3 blockade could be a potential treatment for chronic diseases. However, no functional analysis of these molecules has been reported for cattle and bovine diseases, primarily due to the lack of specific antibodies. Thus, we also investigated the effects of inhibiting the Tim-3/Gal-9 pathway. Predictably, inhibition of the Tim-3/Gal-9 pathway in vitro by Tim-3 expressing cells upregulated the production of IL-2 and IFN-γ. Moreover, combined blockade of PD-L1 and Tim-3 acted in synergy to enhance IFN-γ induction in exhausted cells from BLV-infected cattle. More interestingly, the magnitude to restore cytokine induction was dependent on Tim-3 and Gal-9 expression levels, although a limited number of animals were tested (data not shown). These data, although not showing directly the bindings between Gal-9 derived from the test animals and Tim-3 expressed on transfected cells, might suggest that Tim-3 is involved in the inhibition of T-cell function in BLV infection.

In conclusion, the Tim-3/Gal-9 pathway might link disease progression with T cell exhaustion during BLV infection. Further investigations are required to develop a novel vaccine or therapeutic method against BLV infection, perhaps using anti-Tim-3 antibodies or a recombinant Tim-3 Fc fusion protein.

## Abbreviations

AL, Aleukemic; BLV, Bovine leukemia virus; Con A, Concanavalin A; CTL, Cytotoxic T-lymphocyte; EBL, Enzootic bovine leucosis; Gal-9, Galectin-9; HCV, Hepatitis C virus; HIV, Human immunodeficiency virus; HTLV-1, Human T-cell leukemia virus types 1; IFN, Interferon; IL, Interleukin; LCMV, Lymphocytic choriomeningitis virus; mAb, Monoclonal antibody; PBMCs, Peripheral blood mononuclear cells; PD-1, Programmed death-1; PD-L1, Programmed death-ligand 1; PL, Persistent lymphocytosis; Tim-3, T cell immunoglobulin domain and mucin domain-3.

## Competing interests

The authors declare that they have no competing interests.

## Authors’ contributions

TO carried out all of the studies contained in this manuscript, analyzed data and drafted the manuscript. SK participated in the experimental design, analyzed data, and helped to draft the manuscript. RI participated in some experiments and sample collection. SS participated in some experiments and sample collection. YS participated in the experimental design and reviewed the manuscript. MO helped with experimental design and data interpretation. SM helped in the analysis of the data, overall guidance of the studies. KO supervised the study and reviewed the manuscript. All authors read and approved the final manuscript.
